# Biological Aging Marker p16^INK4a^ in T Cells and Breast Cancer Risk

**DOI:** 10.3390/cancers12113122

**Published:** 2020-10-26

**Authors:** Jie Shen, Renduo Song, Bernard F. Fuemmeler, Kandace P. McGuire, Wong-Ho Chow, Hua Zhao

**Affiliations:** 1 Departments of Epidemiology, The University of Texas MD Anderson Cancer Center, Houston, TX 77030, USA; jieshen@vcuhealth.org (J.S.); rsong@mdanderson.org (R.S.); wchow@mdanderson.org (W.-H.C.); 2 Departments of Family Medicine and Population Health, School of Medicine, Virginia Commonwealth University, Richmond, VA 23284, USA; 3 Health Behavior and Policy, School of Medicine, Virginia Commonwealth University, Richmond, VA 23284, USA; bernard@vcuhealth.org; 4 Department of Surgery, School of Medicine, Virginia Commonwealth University, Richmond, VA 23284, USA; kandace@vcuhealth.org

**Keywords:** p16^INK4a^, breast cancer, stress, biological aging

## Abstract

**Simple Summary:**

The association between cellular senescence, a hallmark of biological aging, and cancer risk has not been examined in population-based studies. To fill the gap, in this study, we assessed the relationship between *p16^INK4a^* mRNA expression in T cells, a marker of cellular senescence, with breast cancer risk and selected sociodemographic and lifestyle variables. Overall, we discovered that higher *p16^INK4a^* mRNA expression in T cells was associated with an increased risk of breast cancer. Also, we found that *p16^INK4a^* mRNA expression in T differed by age, race, family history of cancer, marital status, annual income, and smoking status. The results of this study provide evidence that cellular senescence plays a role in breast cancer development. Furthermore, our results also suggest that social demographics may modify cellular senescence and biological aging.

**Abstract:**

Prior research has demonstrated that altered telomere length, a well-known marker for biological aging, is associated with various types of human cancer. However, whether such association extends to additional hallmarks of biological aging, including cellular senescence, has not been determined yet. In this two-stage study, we assessed the association between *p16^INK4a^* mRNA expression in T cells, a marker of cellular senescence, and breast cancer risk. The discovery stage included 352 breast cancer patients and 324 healthy controls. *p16^INK4a^* mRNA expression was significantly higher in individuals who were older, Black, and had family history of cancer than their counterparts in both cases and controls. *p16^INK4a^* mRNA expression also differed by marital status, annual income, and smoking status in cases. In the discovery stage, we found that increased *p16^INK4a^* mRNA expression was associated with 1.40-fold increased risk of breast cancer (OR = 1.40; 95%CI: 1.21, 1.68; *p* < 0.001). A marginally significant association was further observed in the validation stage with 47 cases and 48 controls using pre-diagnostic samples (OR = 1.28; 95%CI: 0.98, 2.97; *p* = 0.053). In addition, we found that *p16^INK4a^* mRNA expression was higher in tumors with selected aggressive characteristics (e.g., poorly differentiated and large tumors) than their counterparts. In summary, our results demonstrate that higher *p16^INK4a^* mRNA expression in T cells is a risk factor for breast cancer and further support the role of biological aging in the etiology of breast cancer development. Novelty and Impact Statements: The results from this study provide evidence that cellular senescence, a process of biological aging, plays a role in breast cancer etiology. In addition, our results also support that social demographics may modify cellular senescence and biological aging.

## 1. Introduction

A growing literature links stress exposure to the secretion of catecholamines, which can lead to increased production of oxidants and DNA damage [1,2,3,4,5]. DNA damage serves an important role in biological aging as excess levels of DNA damage can initiate cellular senescence [6]. DNA damage can also accelerate the shortening of telomeres, which can result in cellular senescence when telomeres reach a critically short length [7,8,9]. Importantly, the senescent state has been associated with a heightened release of pro-inflammatory factors, which is thought to be a source of the increased inflammation observed with chronological age, and is suspected to contribute to age-related disease [10,11,12,13,14].

Recent research has identified cell cycle inhibitor p16^INK4a^ as one of the most robust indicators of cellular senescence. Expression of p16^INK4a^ in response to DNA damage and cell stress—termed “stress-induced” or premature senescence—evolved as a protective mechanism to prevent the replication of damaged cells that could develop into cancer or other malignancies [10]. However, pervasive cellular senescence via enhanced p16^INK4a^ can itself become damaging and accelerate aging through heightened inflammation and reduced stem cell and tissue function [6,12]. Recent studies with mice demonstrated that removal of p16^INK4a^-positive cells prevented or slowed the deterioration of several tissues and organs, delayed tumor growth [15], and reduced metastasis in mice exposed to cytotoxic cancer treatments [16]. These studies suggest that senescent cells contribute to the promotion and progression of age-related deterioration and tumorigenesis in mice. Furthermore, expression of *p16^INK4a^* is not an epiphenomenon of aging, but appears to play a causal role in the age-associated replicative decline of several tissues, including T-cells [17].

*p16^INK4a^* mRNA expression, which is not detected in young cells, can result in senescent cells that remain indefinitely within tissues [14,18,19,20], and it may potently be activated by stress. For instance, in a recent study, significant increase in *p16^INK4a^* mRNA expression in blood was observed in relation to an increase in chronic stress exposure and daily stress appraisals [21], suggesting that *p16^INK4a^* mRNA, a biomarker of cellular senescence, may be a mechanism by which exposure to stressful life events “get under the skin”. In addition, both extrinsic lifestyle factors, such as smoking and physical inactivity, and common chronic diseases and their treatments, such as with chronic HIV infection, induce p16^INK4a^ expression, thereby promoting cellular senescence [22,23].

In relation to tumor development, loss of p16^INK4a^ is one of the most frequent events in human tumors and allows precancerous lesions to bypass senescence. On the other hand, lasting p16^INK4a^ expression drives cells to enter senescence and thereby aging. Thus, precise regulation of p16^INK4a^ is essential to tissue homeostasis, maintaining a coordinated balance between tumor suppression and aging [24]. To date, the role of cell senescence and *p16^INK4a^* expression in the development of breast cancer has not been evaluated in molecular epidemiologic studies. To fill the gap, we conducted a two-stage study (discovery and validation) to assess the relationship between *p16^INK4a^* mRNA expression in T cells and breast cancer risk. In the discovery stage, we compared *p16^INK4a^* mRNA expression in T cells obtained from breast cancer cases and healthy controls. In the validation stage, we validated the association in a nested breast cancer case–control study using pre-diagnostic peripheral blood mononuclear cells (PBMCs).

## 2. Materials and Methods

### 2.1. Study Population

The study participants in the discovery stage were selected from an ongoing breast cancer case–control study beginning in 2012. Participants were patients at The University of Texas M. D. Anderson Cancer Center (Houston, TX, USA) with newly diagnosed (defined by the presence of malignant breast epithelial cells) and histologically confirmed (by microscopic analysis and molecular subtype) breast cancer. Blood samples were drawn prior to any cancer treatment. Controls were identified largely from female residents of Harris County using random digit dialing. Written informed consent was obtained from each study participant. To assess the relationship between *p16^INK4a^* mRNA expression in T cells and breast cancer risk, we selected 400 cases consecutively recruited since the start of 2015. We reached the goal around June of 2016. During the same period, we also recruited 362 controls. Those cases and controls were included in this study. Self-reported ethnic background was used to define race and ethnicity. The in-person, interviewer-administered questionnaires were conducted at the time of enrollment, which included sociodemographic, reproductive, comorbidities, and other measures. Definitions used in the National Health Interview Survey (NHIS by CDC) were applied to define demographic variables, such as smoking and drinking status and physical activity in the past 12 months. All subjects gave their informed consent for inclusion before they participated in the study. The study was conducted in accordance with the Declaration of Helsinki, and the protocol was approved by Institutional Review Board at M D Anderson Cancer Center.

To validate the results, we ascertained specimens and data from an independent sample of 50 incident breast cancer cases and 50 controls from Mano-A-Mano, the Mexican American Cohort study (MAC). A detailed description of breast cancer cases in the MAC study has been described previously [25,26]. By 1 December 2017, with a median follow-up time of 8.2 years, a total of 126 newly diagnosed breast cancers were identified. Among them, 109 were validated through the Texas Cancer Registry and had blood samples that were collected at baseline. The case selection was based on the availability of PBMC samples in the biorepository. We only selected the cases whose samples were collected at least one year before their cancer diagnosis. The cases and controls were matched on age at recruitment (±2 years) and date of biospecimen collection (±1 year). The study protocol was approved by the Institutional Review Board at M. D. Anderson Cancer Center.

### 2.2. P16^INK4a^ mRNA Expression Analysis

EasySep™ Human T Cell Isolation Kit (Stemcell, Cambridge, MA, USA; Cat#17951) was used to isolate T cells from frozen peripheral blood mononuclear cells. Total RNA was isolated from the isolated T cells by using Trizol reagent (ThermoFisher, Carlsbad, CA, USA; Cat#15596026). RT reactions were conducted using the QuantiTect Reverse Transcription kit (QIAGEN, Germantown, MD, USA; Cat#205311). Expression of *p16^INK4a^* mRNA was quantified by qPCR (standard curve method) using at least two independent RT reactions for each sample and the QuantiNova SYBR^®^ Green PCR Kit (QIAGEN, Germantown, MD, USA; Cat#208052). The following primers were used: (forward) CCAACGCACCGAATAGTTACG, (reverse) GCGCTGCCCATCATCATG. Additionally, 18 s expression was measured as a mean to normalize *p16^INK4a^* levels. The 18 s primers were (forward) TCAACTTTCGATGGTAGTCGCCGT, (reverse) TCCTTGGATGTGGTAGCCGTTTCT. Using this method, 48 cases and 38 controls in the discovery stage, and 3 cases and 2 controls in the validation stage failed analysis due to either insufficient nucleic acid yield, poor quality RNA, or replicate failure. They were excluded from further analysis. We compared the distribution of social demographics, health behaviors, and tumor characteristics between the excluded and included samples. No statistically significant difference was observed in both cases and controls.

### 2.3. Statistical Analysis

We used the statistical software package SAS version 9.4 (SAS, Cary, NC, USA) for all analyses. Because *p16^INK4a^* mRNA expression increases exponentially with age, results were logarithmically transformed. First, we evaluated whether *p16^INK4a^* expression and selected social demographics (age, race, education, marital, income, BMI, and family history of cancer) and healthy behaviors (cigarette smoking, alcohol drinking, physical activity, and sitting time) differed between breast cancer patients and healthy controls. The Student t test was used for two-level dichotomous variables, and analysis of variance was used for variables with more than two levels. Next, we used linear regression analysis to evaluate whether mean *p16^INK4a^* expression differed across categories in each of the selected demographic variables of the cases and controls and tumor characteristics (estrogen receptor (ER) status, tumor stage, grade, and size) of the cases. Age was adjusted in the analysis. We also compared case–control difference in *p16^INK4a^* expression in each category of each selected demographic variable. For the association between *p16^INK4a^* expression and breast cancer risk, we used unconditional multivariate logistic regression to estimate odds ratios (ORs) and 95% Confidence Intervals (CIs). The analysis was adjusted for potential confounders. *p16^INK4a^* expression was treated as a continuous variable or as a categorical variable in dichotomous and quartile analyses. In dichotomized analysis, *p16^INK4a^* expression was designated as “high” or “low” using the controls’ 75% levels of *p16^INK4a^* expression as cutoffs. In quartile analysis, *p16^INK4a^* expression was designated using the controls’ quartile levels of *p16^INK4a^* expression as cutoffs. In the validation analysis, *p16^INK4a^* expression was treated as a continuous variable. We applied similar multivariate logistic regression analysis to assess relationships between *p16^INK4a^* expression and breast cancer risk.

## 3. Results

After excluding samples that failed in *p16^INK4a^* expression analysis (48 cases and 38 controls), a total of 352 breast cancer cases and 324 healthy controls was included in the analysis (Table 1). In terms of social demographics, no significant differences between cases and controls were observed for race, marital status, and BMI category. Compared to the controls, cases were older (56.82% ≥51 years vs. 46.30% ≥51 years) (*p* < 0.006) and a greater percentage had a family history of cancer (18.47% vs. 8.95, *p* < 0.001). A borderline difference between cases and controls was observed for education (*p* = 0.089) and income (*p* = 0.058), with cases trending toward lower educational attainment and income. No significant differences were observed between the groups with respect to smoking status, alcohol use, physical activity, or time sitting. For tumor characteristics, 23.86% cases were estrogen receptor negative (ER-), 19.89% had stage III tumors, 23.58% had poorly differentiated tumors, and 21.31% had large tumors (≥2 cm). Overall, the cases had statistically significantly higher *P16^INK4a^* mRNA expression in T cells than the controls (4.58% vs. 3.27%, *p* < 0.0001).

Next, we assessed the relationship between *p16^INK4a^* mRNA expression and social demographics and lifestyle factors within the controls after adjusting age (Table 2). Compared to younger women (<51 years), older women (≥51 years) had higher *p16^INK4a^* mRNA expression (4.72 vs. 2.02, *p* < 0.001). Compared to White women, Black women had statistically significantly higher *p16^INK4a^* mRNA expression (3.79 vs. 3.08, *p* = 0.021). No statistical significance in *p16^INK4a^* mRNA expression was observed between Hispanic and White women. Compared to those with no family history of cancer, those with family history of cancer had higher *p16^INK4a^* mRNA expression (4.90 vs. 3.11, *p* < 0.001). Furthermore, no significant difference in *p16^INK4a^* mRNA expression was observed across education, marital status, income, BMI category, smoking status, alcohol status, physical activity, and sitting time. The same analysis was also applied to the cases. Similarly, older cases had higher *p16^INK4a^* mRNA expression than younger cases (5.79 vs. 2.99, *p* < 0.001), Black cases had statistically significantly higher *p16^INK4a^* mRNA expression than their White counterparts (5.18 vs. 4.22, *p* = 0.013), and cases with family history of cancer had higher *p16^INK4a^* mRNA expression than those without (5.86 vs. 4.29, *p* < 0.001). Cases who were not married or living together had higher *p16^INK4a^* mRNA expression than those who were married or living together (4.92 vs. 4.27, *p* = 0.029). In addition, we found that cases with less than USD 50,000 annual income had higher *p16^INK4a^* mRNA expression than those with at least USD 50,000 annual income (4.93 vs. 4.25, *p* = 0.009). *p16^INK4a^* mRNA expression was also found diffed by smoking status. Compared to never smokers, current smokers had higher *p16^INK4a^* mRNA expression (5.21 vs. 4.33, *p* = 0.039). In addition, current drinker had marginally significant higher *p16^INK4a^* mRNA expression than never drinkers (4.96 vs. 4.29, *p* = 0.068). We also assessed the relationship between tumor characteristics and *p16^INK4a^* mRNA expression among cases. Higher *p16^INK4a^* mRNA expression was observed in cases with poorly differentiated tumors (*p* = 0.002) and larger (≥2 cm) tumors (*p* = 0.025) than their counterparts. Then, we assessed whether higher *p16^INK4a^* mRNA expression differed between cases and controls in each category of selected characteristics. As expected, the cases had statistically significantly higher *p16^INK4a^* mRNA expression than the controls in each category, except with family history of cancer (*p* = 0.280).

We then examined the association between higher *p16^INK4a^* mRNA expression in T cells and breast cancer risk (Table 3). If treated as a continuous variable, increased higher *p16^INK4a^* mRNA expression was associated with 1.40-fold increased risk of breast cancer after adjusting age, race, education, marital, income, BMI category, family history of cancer, smoking status, alcohol status, physical activity, and sitting time (OR = 1.40; 95%CI: 1.21, 1.68; *p* < 0.001). In dichotomized analysis, using the 75% levels of *p16^INK4a^* mRNA expression in controls as the cutoff point (4.76), those with higher *p16^INK4a^* mRNA expression had 1.81-fold increased risk of breast cancer (OR = 1.81; 95%CI: 1.29, 2.45; *p* < 0.001). In further quartile analysis, the risk association between increased *p16^INK4a^* mRNA expression and breast cancer risk was further validated. Compared to those who had the lowest (1st quartile) *p16^INK4a^* mRNA expression, those with highest (4th quartile) *p16^INK4a^* mRNA expression had 2.46-fold increased risk of breast cancer (OR = 2.46; 95%CI: 1.57, 4.04; *p* < 0.001). In addition, a significant trend of increasing risk of breast cancer was observed when *p16^INK4a^* mRNA expression increased (*p* < 0.001).

Finally, we attempted to confirm the observed significant association between *p16^INK4a^* mRNA expression and breast cancer risk in pre-diagnostic PBMCs (Table 4). The cases and controls were well-matched on age, parity, education level, birthplace, language acculturation, BMI category, smoking status, alcohol drinking, and physical activity. Compared to healthy controls (*n* = 48), incident breast cancer cases (*n* = 47) had statistically significant higher levels of *p16^INK4a^* mRNA expression (4.39 vs. 3.41, *p* = 0.037). In the univariate analysis, higher *p16^INK4a^* mRNA expression in PBMCs was associated with 1.29-fold increased risk of breast cancer (OR = 1.29; 95%CI: 1.02, 2.72, *p* = 0.047). In the multivariate analysis, higher *p16^INK4a^* mRNA expression was marginally associated with 1.28-fold increased risk of breast cancer (OR = 1.28; 95%CI: 0.98, 2.97; *p* = 0.053) after adjusting age, BMI category, smoking status, alcohol status, and physical activity.

## 4. Discussion

To date, no study has evaluated the association between *p16^INK4a^* mRNA expression in T cells and breast cancer risk. In the discovery phase using 48 breast cancer cases and 47 controls, we found that increased pre-treatment *p16^INK4a^* mRNA expression was associated with 1.40-fold increased risk of breast cancer (OR = 1.40; 95%CI: 1.21, 1.68; *p* < 0.001). A marginally significant association was further observed in the validation stage using pre-diagnostic blood samples from the Mano-A-Mano cohort, as increased *p16^INK4a^* mRNA expression was associated with 1.28-fold increased risk of breast cancer (OR = 1.28; 95%CI: 0.98, 2.97; *p* = 0.053). In addition, we found that *p16^INK4a^* mRNA expression differed by age, race, and family history of cancer in both case and control groups, and by marital status, annul income, and smoking status in the case group. In addition, we found that *p16^INK4a^* mRNA expression was higher in tumors with selected aggressive characteristics (e.g., poorly differentiated and large tumors) than their counterparts.

The significant association between age group and *p16^INK4a^* mRNA expression is expected since *p16^INK4a^* mRNA expression is a marker for cell senescence, which is associated with biological aging [24]. We observed that Black women had higher *p16^INK4a^* mRNA expression than White women in our study in both cases and controls. Though racial difference between Black and White women in telomere length, the best known marker of biological aging, has been reported previously [27,28,29,30], no study has reported the racial difference in *p16^INK4a^* mRNA expression. In telomere length, most of the studies have found that Black and/or Hispanic women had shorter telomere length than White women [27,28,30]. Furthermore, the rate of telomere shortening, which may reflect the cumulative burden of exposure to various chronic stressors over the life course, was found quicker in Black and/or Hispanic women than White women [27,28,30]. Those findings support the notion that exposure to adverse social conditions (e.g., racism) is associated with accelerated biological aging [31]. In fact, in the United States, compared to White women, Black and Hispanic women are more likely to exposure to higher levels of social adversity during their lifetime [32,33,34]. The cumulative exposure to higher life-course adversity among Black and Hispanic women may therefore increase the likelihood of accelerated biological aging and displaying aging phenotypes, cellular senescence with shortened telomere and elevated *p16^INK4a^* mRNA expression, and ultimately increase their risks of breast cancer, developing more aggressive breast tumor phenotypes, and shortened survival [35].

In support of this hypothesis, in this study, we found that breast cancer cases with less than USD 50,000 annual income had higher *p16^INK4a^* mRNA expression than those with at least USD 50,000 annually (*p* = 0.009). A similar trend was also observed for education, with lower education having higher *p16^INK4a^* mRNA expression, but the difference did not reach statistical significance. Interestingly, we also found *p16^INK4a^* mRNA expression was higher in breast cancer cases who were not married or living together than cases who were married or living together (*p* = 0.029). Social support is arguably the fundamental cause of health differentials. The mutual support from the family member and/or partner will provide a buffer that can help better weather adverse social conditions and reduce stress, which, consequently, may slow down the biological aging process. To date, only one study has assessed the relationship between social adversity, chronic stress, and *p16^INK4a^* mRNA expression [21], which shows that chronic stress exposure and daily stress appraisals were associated with increased *p16^INK4a^* mRNA expression. Our results may suggest that exposure to adverse social conditions is associated with accelerated biological aging, offering one mechanism through which adversity may increase the risk for age-related diseases, such as breast cancer. 

We also observed that *p16^INK4a^* mRNA expression could be modified by cigarette smoking status. Our results are consistent with previous findings [22,36,37]. Liu et al. reported that dosage effect as *p16^INK4a^* expression in peripheral blood T-cells was associated with cumulative exposure as estimated by tobacco pack-years [22]. It has been reported that DNA damage from cigarette smoke induces senescence via the p16 pathway, and targeting p16-induced senescence could prevent cigarette smoking-induced emphysema in mice [36]. The study by Liu et al. also reported an inverse relationship between exercise and *p16^INK4a^* mRNA expression [22]. In our study, we found that those with medium or high levels of physical activity had lower *p16^INK4a^* mRNA expression in both case and control groups. However, none of the association reached statistical significance (*p* = 0.124 and 0.189, respectively). We also failed to observe the association between sitting time and *p16^INK4a^* mRNA expression. However, similar to Liu’s study, no significant relationship between obesity and *p16^INK4a^* mRNA expression was found. One interesting observation in our study is that the difference in *p16^INK4a^* mRNA expression by income level, marital status, and smoking status was more evident in breast cancer cases than controls. It is possible that there is not enough variation in those social demographics and healthy behaviors in our controls. It may also suggest that cancer diagnosis may have an influence. Thus, in the future, large prospective studies are needed to further clarify the relationship.

The higher *p16^INK4a^* mRNA expression in both cases and controls with family history of cancer than those without is intriguing. The experience of immediate family members with cancer is a life stressor to their relatives which trigger different cognitions that determine whether they will suffer from cancer by heredity in the future, leading to different coping styles and different psychological reactions [38,39]. It has been reported that the cancer-specific distress among women with a family history of breast cancer was higher than that among women without a family history [40,41]. A previous study has suggested that positive coping style was associated with good psychological adjustment, and negative coping style was related to maladjustment and was harmful to individual psychological health [42,43]. Unfortunately, the information on coping style is not available in our study.

The association between higher *p16^INK4a^* mRNA expression and breast cancer risk is expected. Expression of p16^INK4a^ in response to accelerated biological aging and cell senescence is originally aimed to be a protective mechanism to prevent the replication of damaged cells from developing into cancer or other malignancies [6,10]. However, pervasive cellular senescence via enhanced p16^INK4a^ expression can itself become damaging and accelerate biological aging because some senescent cells will secrete molecules including pro-inflammatory cytokines, growth factors, and matrix-remolding enzymes [12,44]. Those resulting pro-inflammatory cytokines could summon inflammatory cells and promote growth and survival of nearly cells. In the case of breast carcinogenesis, if breast premalignant and/or tumor cells are nearby, those pro-inflammatory cytokines will contribute to the promotion and progression of breast tumor. In our study, the association between *p16^INK4a^* mRNA expression and breast cancer risk was weakened when using pre-diagnostic samples. This may be simply because of the smaller sample size which did not provide adequate statistical power for us to detect the association. It may also suggest that *p16^INK4a^* mRNA expression differs by the breast carcinogenesis process. It has been suggested that *p16^INK4a^* mRNA expression is increased in pre-malignant lesions but decreased after tumor development [45,46,47]. All pre-diagnosed samples from the breast cancer cases were obtained from at least one year prior to the date of disease diagnosis, but with a wide range of from 1 to 15 years. The sample size is too small to be further stratified by the duration between blood drawn and disease diagnosis. We also did not have the information in this study to determine when the pre-malignant lesions and tumors actually began to develop, thus, the variation of *p16^INK4a^* mRNA expression by breast carcinogenesis process cannot be accounted for in our analyses. Another possibility is the difference in biospecimens used in analyzing *p16^INK4a^* mRNA expression, T cells in the discovery study, and PBMC in the validation study. In addition to T cells, PBMCs contain other lymphocytes (e.g., B cells and NK cells). Though both T cells and PBMCs have been used in studying *p16^INK4a^* mRNA expression [21,22,23], it is possible that the relationship observed in T cells may be weakened in PBMCs. 

## 5. Conclusions

In summary, we have demonstrated that increased *p16^INK4a^* mRNA expression in T cells is associated with increased risk of breast cancer. We also reported that *p16^INK4a^* mRNA expression differed by selected social demographics, healthy behaviors, and tumor characteristics. Due to the modest sample size, particularly in validation stage, our results need to be further validated in large prospective cohort studies. Yet, the results from this study lend a support to the assumption that chronic stress is associated with accelerated aging by inducing cellular senescence, consequently contributing to increased risk of breast cancer among women. 

## Figures and Tables

**Table 1:** Distribution of characteristics among participants by case–control status.

Variable	Controls, *n* (%)	Cases, *n* (%)	*p* Value
Overall	324 (100)	352 (100)	
*P16^INK4a^*, mean (SD)	3.27 (2.31)	4.58 (2.47)	<0.001
Age (by median in controls)			
<51 years	174 (53.70)	152 (43.18)	
≥51 years	150 (46.30)	200 (56.82)	0.006
Race			
White	192 (59.26)	212 (60.23)	
Black	89 (27.47)	96 (27.27)	
Hispanic	43 (13.27)	44 (12.50)	0.948
Education			
<college	129 (39.81)	163 (46.31)	
≥some college	195 (60.19)	189 (53.69)	0.089
Marital status			
Married or living together	171 (52.78)	184 (52.27)	
Other	153 (47.22)	168 (47.73)	0.896
Income			
<USD 50,000	133 (41.05)	170 (48.30)	
≥USD 50,000	191 (58.95)	182 (51.70)	0.058
BMI category			
Underweight/normal weight	90 (27.78)	82 (23.30)	
Overweight	149 (45.99)	167 (47.44)	
Obese	85 (26.23)	103 (29.26)	0.374
Family history of cancer			
No	295 (91.05)	287 (81.53)	
Yes	29 (8.95)	65 (18.47)	<0.001
Smoking status			
Never	173 (53.40)	166 (47.16)	
Former	92 (28.40)	108 (30.68)	
Current	59 (18.21)	78 (22.16)	0.234
Alcohol drinking			
Never	158 (48.77)	153 (43.47)	
Former	69 (21.30)	87 (24.72)	
Current	97 (29.94)	112 (31.82)	0.354
Physical activity			
Low	172 (53.09)	180 (51.14)	
Medium or high	152 (46.91)	172 (48.86)	0.612
Sitting time			
<4 h/day	159 (49.07)	162 (46.02)	
≥4 h/day	165 (50.93)	190 (53.98)	0.427
Tumor subtype			
ER+		268 (76.14)	
ER−		84 (23.86)	
Tumor stage			
I/II		282 (80.11)	
III		70 (19.89)	
Tumor grade			
Well/moderate differentiated		269 (76.42)	
Poorly differentiated		83 (23.58)	
Tumor size			
<2 cm		277 (78.69)	
≥2 cm		75 (21.31)	

**Table 2:** Comparison of *P16^INK4a^* expression by demographics and tumor characteristics.

Variable	Mean (SD)	*p* Value *	Mean (SD)	*p* Value *	*p* Value ^$^
	Controls		Cases		
Age at enrollment, years (by median in control)					
<51 years	2.02 (1.79)	1.000	2.99 (1.78)	1.000	<0.001
≥51 years	4.72 (2.76)	<0.001	5.79 (2.57)	<0.001	<0.001
Race					
White	3.08 (2.12)	1.000	4.22 (2.93)	1.000	<0.001
Black	3.79 (3.01)	0.021	5.18 (3.79)	0.013	0.008
Hispanic	3.04 (2.55)	0.925	5.01 (4.76)	0.180	0.021
Education					
<College	3.11 (2.50)	1.000	4.39 (2.88)	1.000	<0.001
≥Some college	3.38 (2.14)	0.327	4.74 (2.46)	0.204	<0.001
Marital status					
Married or living together	3.06 (2.48)	1.000	4.27 (2.73)	1.000	<0.001
Others	3.50 (2.61)	0.134	4.92 (2.77)	0.029	<0.001
Income					
<USD 50,000	3.34 (2.58)	1.000	4.93 (2.39)	1.000	<0.001
≥USD 50,000	3.22 (2.49)	0.662	4.25 (2.28)	0.009	<0.001
BMI category					
Under/normal weight	3.22 (2.71)	1.000	4.39 (2.56)	1.000	0.006
Overweight	3.30 (2.44)	0.826	4.48 (2.31)	0.790	<0.001
Obese	3.27 (2.82)	0.911	4.89 (2.72)	0.229	<0.001
Family history of cancer					
No	3.11 (2.19)	1.000	4.29 (2.31)	1.000	<0.001
Yes	4.90 (3.21)	<0.001	5.86 (3.82)	<0.001	0.280
Smoking status					
Never	3.20 (2.56)	1.000	4.33 (2.82)	1.000	<0.001
Former	3.25 (3.26)	0.904	4.51 (2.62)	0.612	0.007
Current	3.51 (2.62)	0.471	5.21 (3.39)	0.039	0.006
Alcohol drinking					
Never	3.18 (2.37)	1.000	4.29 (2.87)	1.000	<0.001
Former	3.26 (3.02)	0.843	4.60 (3.13)	0.503	0.011
Current	3.42 (3.16)	0.507	4.96 (2.79)	0.068	<0.001
Physical activity					
Low	3.44 (2.32)	1.000	4.77 (2.31)	1.000	<0.001
Medium or high	3.08 (2.47)	0.189	4.38 (2.37)	0.124	<0.001
Sitting time					
<4 h/day	3.12 (2.56)	1.000	4.40 (2.84)	1.000	<0.001
≥4 h/day	3.41 (2.49)	0.326	4.73 (2.55)	0.279	<0.001
Tumor subtype					
ER+			4.47 (2.26)	1.000	
ER−			4.93 (4.01)	0.198	
Tumor stage					
I/II			4.55 (2.38)	1.000	
III			4.70 (3.89)	0.714	
Tumor grade					
Well/moderate differentiated			4.32 (2.19)	1.000	
Poorly differentiated			5.42 (3.47)	0.002	
Tumor size					
<2 cm			4.41 (2.29)	1.000	
≥2 cm			5.21 (3.55)	0.025	

*: Comparison within case and control groups, adjusted by age if appropriate, ^$^: comparison between case and control groups, adjusted by age if appropriate.

**Table 3:** Association between *P16^INK4a^* expression and breast cancer risk in the case–control study.

*p16^INK4a^* Expression	Controls, N (%)	Cases, N (%)	Unadj. OR (95%CI)	*p* Value	Adj. OR (95% CI) *	*p* Value
Continuous (0.1% unit)	324 (100)	352 (100)	1.40 (1.21, 1.68)	<0.001	1.36 (1.19, 1.58)	<0.001
By 75% in controls						
<4.76	244 (75.31)	213 (60.51)	Reference		Reference	
≥4.76	80 (24.69)	139 (39.49)	1.99 (1.41, 2.81)	<0.001	1.81 (1.29–2.45)	<0.001
By quartile in the controls						
1st	80 (24.69)	52 (14.77)	Reference		Reference	
2nd	82 (25.31)	75 (21.31)	1.41 (0.86, 2.31)	0.153	1.33 (0.80, 2.14)	0.194
3rd	79 (24.38)	86 (24.43)	1.67 (1.03, 2.74)	0.029	1.56 (0.94–2.66)	0.098
4th	83 (25.62)	139 (39.49)	2.58 (1.62, 4.11)	0.010	2.46 (1.57–4.04)	<0.001
*p* for trend				<0.001		<0.001

* Adjusted by age, race, education, marital, income, BMI category, family history of cancer, smoking status, alcohol status, physical activity, and sitting time.

**Table 4:** Validation of the association using pre-diagnostic PBMCs.

*P16^INK4a^* Expression	Controls, N = 47	Cases, N = 48	*p* Value	Unadj. OR (95%CI)	*p* Value	Adj, OR (95% CI) *	*p* Value
Continuous, Mean (SD)	3.41 (2.99)	4.39 (3.08)	0.037	1.29 (1.02, 2.72)	0.047	1.28 (0.98, 2.97)	0.053

* Adjusted by age, education, marital, income, BMI category, family history of cancer, smoking status, alcohol status, physical activity, and sitting.
