# Correction: Shen, J.; et al. Biological Aging Marker p16^INK4a^ in T Cells and Breast Cancer Risk. *Cancers* 2020, *12*, 3122

**DOI:** 10.3390/cancers13020329

**Published:** 2021-01-17

**Authors:** Jie Shen, Renduo Song, Bernard F. Fuemmeler, Kandace P. McGuire, Wong-Ho Chow, Hua Zhao

**Affiliations:** 1 Departments of Epidemiology, The University of Texas MD Anderson Cancer Center, Houston, TX 77030, USA; jieshen@vcuhealth.org (J.S.); rsong@mdanderson.org (R.S.); wchow@mdanderson.org (W.-H.C.); 2 Departments of Family Medicine and Population Health, School of Medicine, Virginia Commonwealth University, Richmond, VA 23284, USA; 3 Health Behavior and Policy, School of Medicine, Virginia Commonwealth University, Richmond, VA 23284, USA; bernard@vcuhealth.org; 4 Department of Surgery, School of Medicine, Virginia Commonwealth University, Richmond, VA 23284, USA; kandace@vcuhealth.org

The authors wish to make the following corrections to this paper [1]: 

An incorrect draft version was inadvertently submitted to the journal. The corrected version is now provided. 

The first two paragraphs in the Introduction section should be revised to:

Elevated production of stress hormones due to stress exposure can increase DNA damage [1,2]. Excessive DNA damage can initiate cellular senescence and further accelerate biological aging [3]. The cell cycle inhibitor p16INK4a is a well-known biomarker for cellular senescence. The expression of p16INK4a due to stress exposure and DNA damage can prevent the replication of cells with severe DNA damage [4]. However, persistent cellular senescence via heightened p16INK4a can become detrimental because certain senescent cells may release pro-inflammatory factors to promote inflammation, damage nearby cells and tissues, further accelerate biological aging, and consequently increase the risk of age-related diseases [3,5]. Intriguingly, studies in mice have shown that eliminating p16INK4a-positive cells not only reduced cellular aging but also hindered tumor growth and reduced tumor progression [6]. This suggests that senescent cells play an essential role in age-related deterioration and tumorigenesis. Furthermore, the expression of p16INK4a is not an epiphenomenon of aging but appears to play a causal role in the age-associated replicative decline of several tissues, including T-cells [7]. 

The updated references in this paragraph are as below:

1. Flint, M.S.; Baum, A.; Chambers, W.H.; Jenkins, F.J. Induction of DNA damage, alteration of DNA repair and transcriptional activation by stress hormones. *Psychoneuroendocrinology* **2007**, *32*, 470–479. Epub 27 April 2007. doi: 10.1016/j.psyneuen.2007.02.013.
2. Hara, M.R.; Kovacs, J.J.; Whalen, E.J.; Rajagopal, S.; Strachan, R.T.; Grant, W.; Towers, A.J.; Williams, B.; Lam, C.M.; Xiao, K.; et al. A stress response pathway regulates DNA damage through beta2-adrenoreceptors and beta-arrestin-1. *Nature* **2011**, *477*, 349–353. doi: 10.1038/nature10368.
3. Campisi, J. Senescent cells, tumor suppression, and organismal aging: good citizens, bad neighbors. *Cell* **2005**, *120*, 513–522. doi: 10.1016/j.cell.2005.02.003.
4. Campisi, J.; d’Adda, di Fagagna F. Cellular senescence: When bad things happen to good cells. *Nat. Rev. Mol. Cell Biol.* **2007**, *8*, 729–740. doi: 10.1038/nrm2233.
5. Coppe, J.P.; Desprez, P.Y.; Krtolica, A.; Campisi, J. The senescence-associated secretory phenotype: The dark side of tumor suppression. *Annu. Rev. Pathol.* **2010**, *5*, 99–118. doi: 10.1146/annurev-pathol-121808-102144.
6. Baker, D.J.; Wijshake, T.; Tchkonia, T.; LeBrasseur, N.K.; Childs, B.G.; van de Sluis, B.; Kirkland, J.L.; van Deursen, J.M. Clearance of p16Ink4a-positive senescent cells delays ageing-associated disorders. *Nature* **2011**, *479*, 232–236. doi: 10.1038/nature10600.
7. Liu, Y.; Johnson, S.M.; Fedoriw, Y.; Rogers, A.B.; Yuan, H.; Krishnamurthy, J.; Sharpless, N.E. Expression of p16(INK4a) prevents cancer and promotes aging in lymphocytes. *Blood* **2011**, *117*, 3257–3267. doi: 10.1182/blood-2010-09-304402.

The fifth paragraph in the Discussion section should be revised to:

The higher levels of *p16INK4a* mRNA expression in both cases and controls with a family history of cancer than those without are intriguing. Learning that a family member has cancer is a stressful event because it may unavoidably lead to the speculation about whether they will also have cancer due to their shared genetic background [29,30]. Previous studies in breast cancer have shown that women with a family history of breast cancer have higher levels of cancer-specific distress than those without a family history [31,32]. A positive coping style can encourage good psychological adjustment and thereby alleviate the stress. On the other hand, a negative coping style can further exacerbate stress and consequently lead to harmful health impacts [33,34]. Unfortunately, the current study did not collect data on coping styles.

The first three sentences of the sixth paragraph in the Discussion section should be revised to:

The relationship between higher *p16INK4a* mRNA expression and breast cancer risk is expected. As mentioned previously, the expression of p16INK4a is a protective mechanism to guard against excessive DNA damage and prevent damaged cells from proliferating and causing further transformation to malignancy [3,4]. However, persistently elevated *p16INK4a* mRNA expression may have a detrimental consequence. Specific senescent cells may secrete pro-inflammatory cytokines, growth factors, and matrix-remolding enzymes that can cause damage to nearby cells or tissues and further promote tumorigenesis [5,35].

Since the number of cited references in the first two paragraphs are changed from 17 to 7, and one within the 17 but not the 7 is cited elsewhere in the paper, the total reference number is thus changed from 47 to 38. Therefore, the remaining reference numbers have been updated to 8–38. 

The authors apologize for any inconvenience caused and state that the scientific conclusions are unaffected. The original article has been updated.
